# Benign and malignant diagnosis of spinal tumors based on deep learning and weighted fusion framework on MRI

**DOI:** 10.1186/s13244-022-01227-2

**Published:** 2022-05-10

**Authors:** Hong Liu, Menglei Jiao, Yuan Yuan, Hanqiang Ouyang, Jianfang Liu, Yuan Li, Chunjie Wang, Ning Lang, Yueliang Qian, Liang Jiang, Huishu Yuan, Xiangdong Wang

**Affiliations:** 1 Beijing Key Laboratory of Mobile Computing and Pervasive Device, Institute of Computing Technology, Chinese Academy of Sciences, No. 6 Kexueyuan South Road, Haidian District, Beijing, 100190 China; 2 Department of Radiology, Peking University Third Hospital, 49 North Garden Road, Haidian District, Beijing, 100191 China; 3 Department of Orthopaedics, Peking University Third Hospital, 49 North Garden Road, Haidian District, Beijing, 100191 China; 4 Engineering Research Center of Bone and Joint Precision Medicine, Beijing, 100191 China; 5 Beijing Key Laboratory of Spinal Disease Research, Beijing, 100191 China; 6 University of Chinese Academy of Sciences, Beijing, 100086 China

**Keywords:** Spine tumor, Benign, Malignant, Deep learning, MRI

## Abstract

**Background:**

The application of deep learning has allowed significant progress in medical imaging. However, few studies have focused on the diagnosis of benign and malignant spinal tumors using medical imaging and age information at the patient level. This study proposes a multi-model weighted fusion framework (WFF) for benign and malignant diagnosis of spinal tumors based on magnetic resonance imaging (MRI) images and age information.

**Methods:**

The proposed WFF included a tumor detection model, sequence classification model, and age information statistic module based on sagittal MRI sequences obtained from 585 patients with spinal tumors (270 benign, 315 malignant) between January 2006 and December 2019 from the cooperative hospital. The experimental results of the WFF were compared with those of one radiologist (D1) and two spine surgeons (D2 and D3).

**Results:**

In the case of reference age information, the accuracy (ACC) (0.821) of WFF was higher than three doctors’ ACC (D1: 0.686; D2: 0.736; D3: 0.636). Without age information, the ACC (0.800) of the WFF was also higher than that of the three doctors (D1: 0.750; D2: 0.664; D3:0.614).

**Conclusions:**

The proposed WFF is effective in the diagnosis of benign and malignant spinal tumors with complex histological types on MRI.

## Key points

- WFF automatically detects spinal tumors from MRI for patient-level diagnosis.
- Including age information into the AI model can improve diagnostic accuracy.
- The model showed a higher accuracy of diagnosis than doctors.
- WFF showed lower error rate for most tumor locations compared with doctors.

## Background

Spinal tumors include both primary and metastatic tumors. Metastatic spinal tumors are usually malignant, while primary spinal tumors can be further divided into benign and malignant tumors. If benign tumors are not diagnosed in time, they may cause local damage and show invasive growth into other surrounding tissues, whereas malignant tumors may cause systemic multisystem metastasis and threaten the safety of patients. Use of magnetic resonance imaging (MRI) for patients in the early stage has shown great clinical significance in diagnosis of benign and malignant spine tumors.

With the development of deep learning technology, an increasing number of researchers have applied it in the field of medicine, including tumor segmentation [1, 2], detection [3, 4], and classification [5, 6]. However, most of these methods are based on a single image or sequence, and rarely use multiple sequences for patient-level diagnosis, or refer to clinical information.

In clinical practice, doctors usually locate the tumor region first and then make a decision according to multiple images or sequences, along with the clinical information of the patient. Inspired by the diagnostic process of doctors, this study proposes a multi-model weighted fusion framework (WFF) for the diagnosis of benign and malignant spine tumors at the patient level, which includes a tumor detection model, sequence classification model, and an age information statistic module. WFF can automatically locate the tumor region in MRI images, combine the rough classification results of the tumor detection model with the fine classification results of the sequence classification model, aggregate the results of different sequences by majority voting, and refer to the patient’s age information simultaneously for patient-level diagnosis.

## Materials and methods

### Image data

The final pathological diagnosis reports of consecutive patients with spinal tumors visiting the cooperative hospital between January 2006 and December 2019 were retrospectively reviewed with approval from the Institutional Review Board (IRB). This study included sagittal MRI images collected from 585 patients with spinal tumors (259 women, 326 men; mean age 48 ± 18 years, range 4–82 years), including 270 benign and 315 malignant patients. All patients had definite pathological results confirmed by trocar biopsy or surgery and were divided into a training set (*n* = 445; 180 benign, 265 malignant) and a testing set (*n* = 140; 90 benign, 50 malignant), as shown in Table 1. The training set included metastases and primary spinal tumors, whereas the testing set only included primary spinal tumors. There were 2150 sequences obtained from 585 patients, including 1625 sequences for training and 525 sequences for testing, and the slice thickness ranged from 3 to 7 mm. Each patient underwent T1 (T1WI) and T2 (T2WI, FS-T2WI) sequences. Four radiologists and one spine surgeon annotated the tumor regions of these images with rectangles using LabelMe [7] and checked the labeled regions with each other to ensure reliability. There were 20,593 annotated images, of which 15,778 were for training and 4815 for testing. Each patient had an average of four sequences, and each sequence had an average of nine labeled images. The benign and malignant regions of these annotated tumor regions were determined based on the patient's pathological report.

**Table 1:** The details of spinal tumor dataset

Tumor type	Training set	Test set
Benign	180 patients/5177 images	90 patients/2576 images
Malignant	265 patients/10601 images	50 patients/2239 images
Total	445 patients/15778 images	140 patients/4815 images

**Fig. 1:**
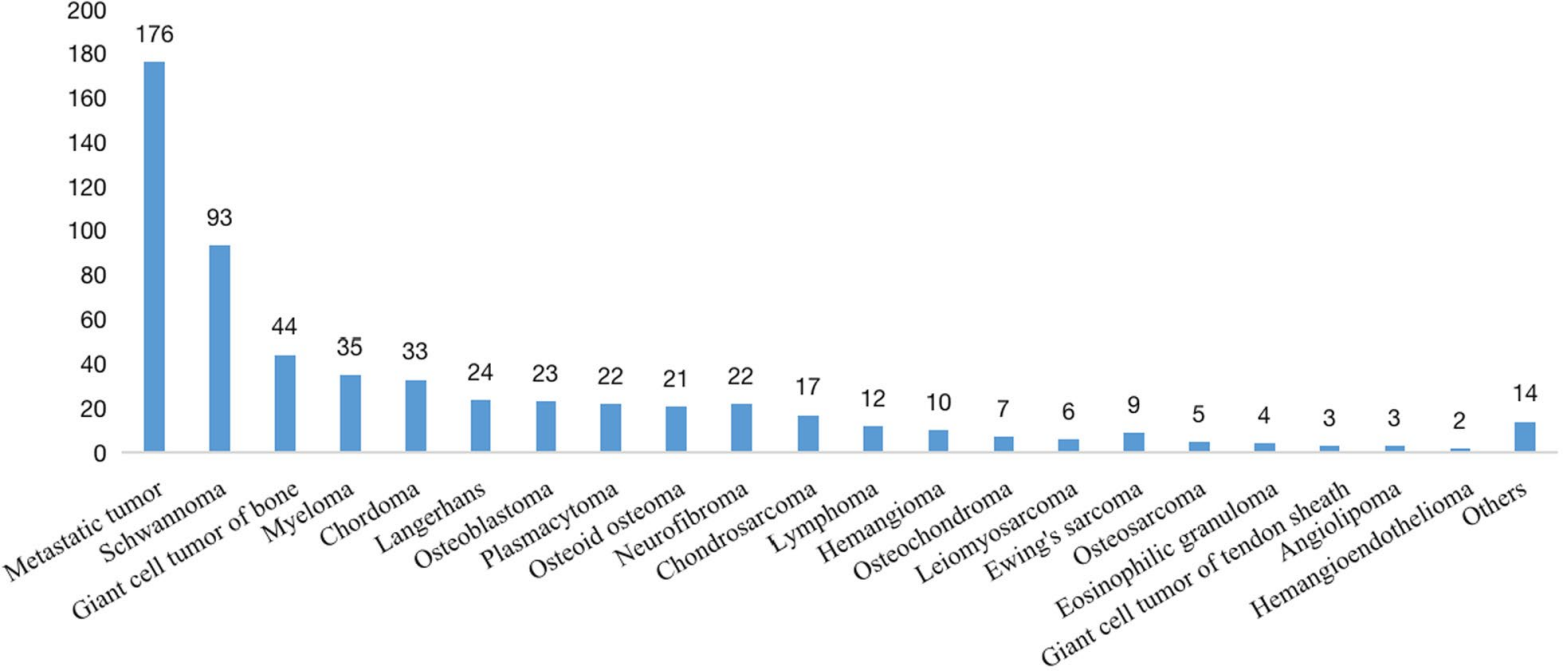
Pathological distribution of all patients

Our dataset is a complex spinal tumor dataset with more than 20 histological subtypes, as shown in Fig. 1. It should be noted that our cooperative hospital is the largest spine tumor center in our country, which has received a large number of spine tumor referrals and has performed a large number of spine tumor operations every year. Therefore, our focus included spinal tumors and some neurogenic tumors that extend to or affect the spine structure (such as schwannoma and neurofibroma) [8, 9], and intradural and intramedullary tumors were further referred to the Department of Neurosurgery. The tumors were located in different vertebrae, including the cervical, thoracic, lumbar, and sacral vertebrae, as shown in Table 2. Diagnosing such a complex spinal tumor dataset is challenging.

**Table 2:** Number of cases corresponding to tumor location

Tumor location	Cervical vertebra	Thoracic vertebra	Lumbar vertebra	Sacral vertebra
Number of cases	297	182	174	24
Total	677			

### Proposed framework

**Fig. 2:**
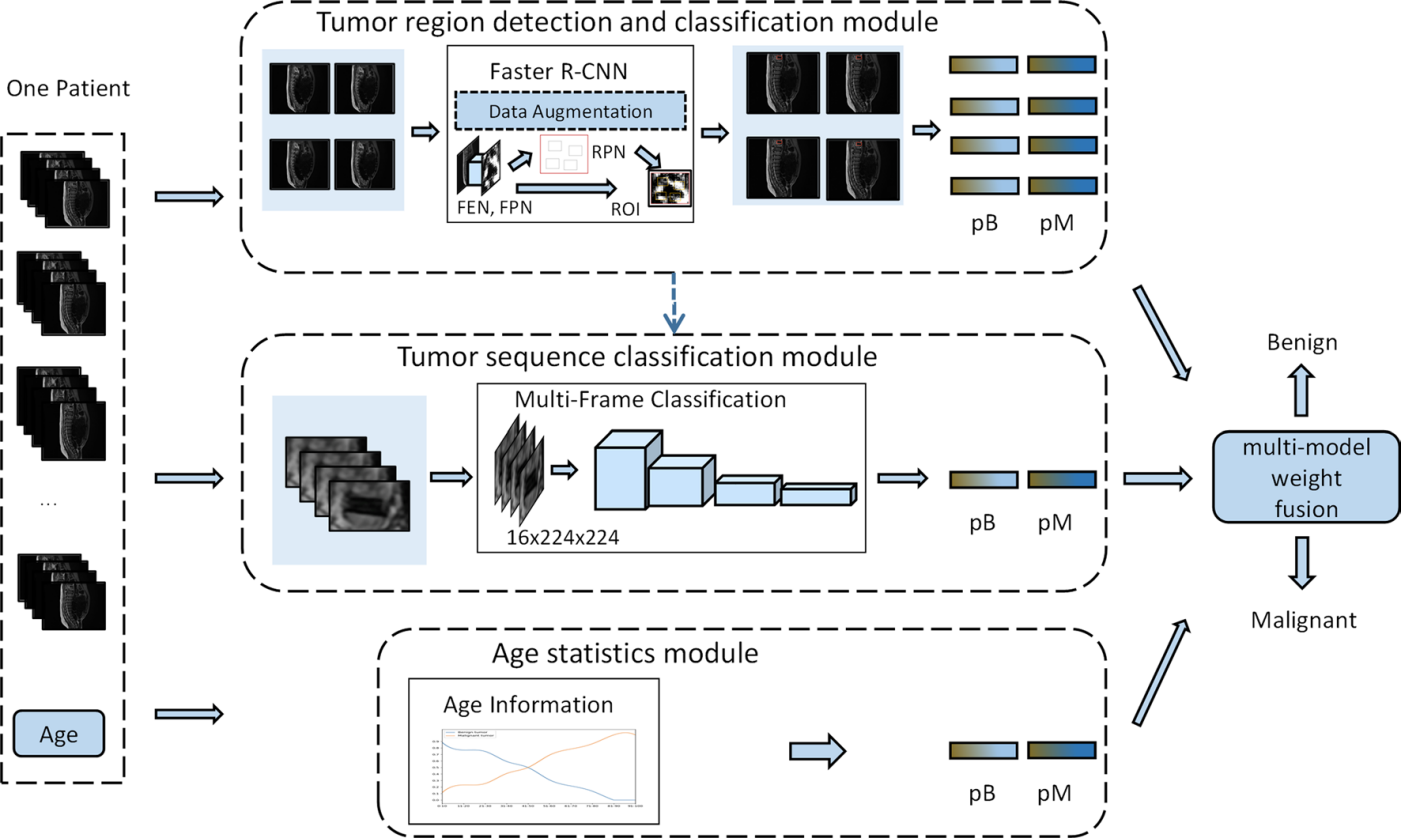
The proposed multi-model weighted fusion framework (WFF)

This study proposes a multi-model weighted fusion framework (WFF) based on sagittal MRI sequences, which can combine the tumor detection model, sequence classification model, and age information statistic module to diagnose benign and malignant spinal tumors at the patient level, as shown in Fig. 2, where ${p}_{b}$ and ${p}_{m}$ in Fig. 2 represent the probability of benign and malignant tumors, respectively. First, we used Faster-RCNN [10] to detect the tumor region in each MRI image and provide a rough probability of being benign or malignant. Subsequently, a sequence classification model was applied to classify the detected tumor regions to obtain sequence-level results. Finally, a weighted fusion decision was made according to the results of the above two models and age information for the final diagnostic results. Four-fold cross-validation was applied to the training set to train and validate the WFF, and the appropriate hyperparameters of the deep models and fused weights were selected.

### Detection model for tumor localization and rough classification

**Fig. 3:**
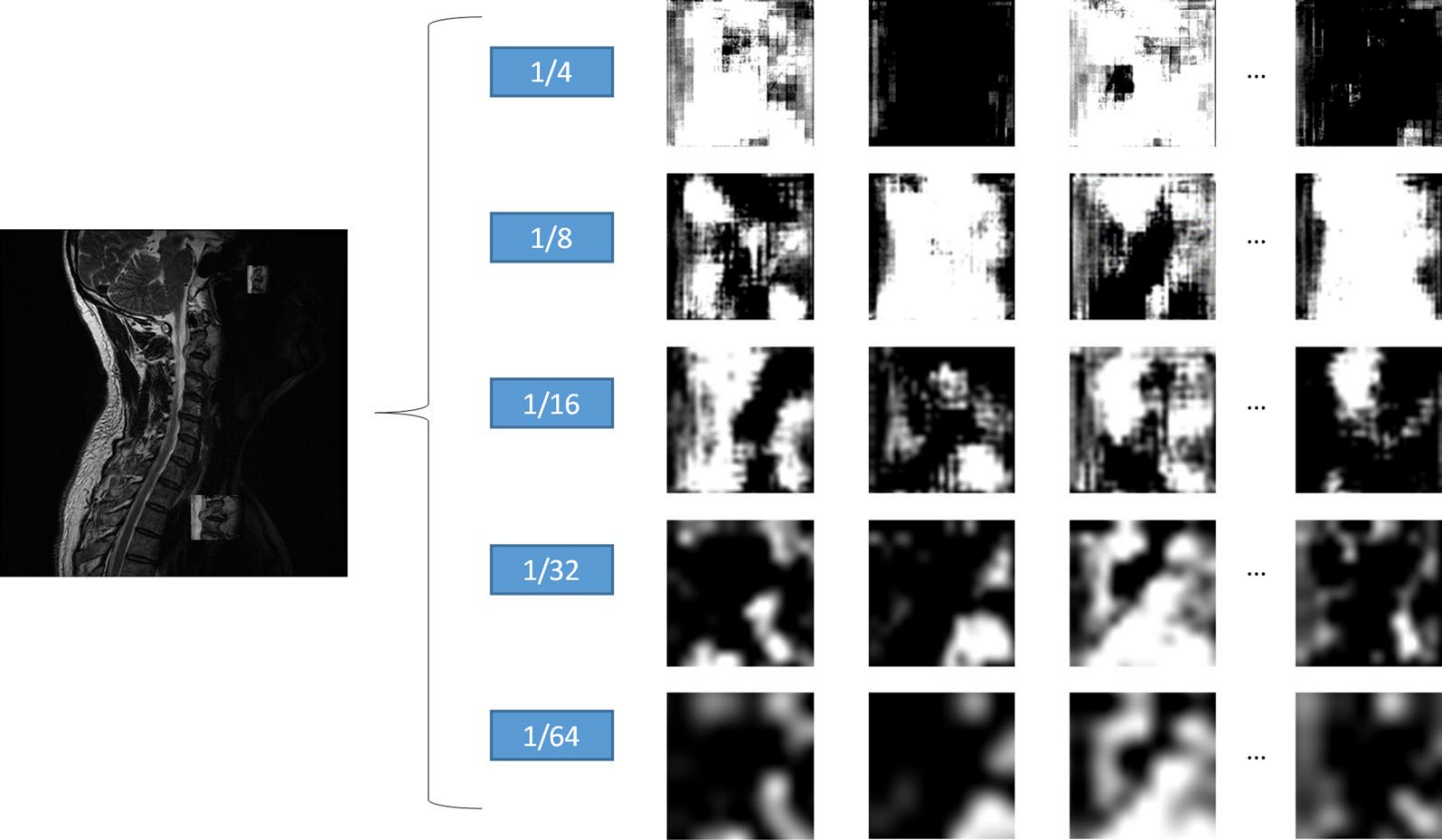
Feature maps extracted with five scales

This study used a Faster-RCNN with tri-class as the tumor detection model. With the limited labeled tumor regions, the MultiScale-SelfCutMix method [11] was used for data augmentation, which randomly extracts the labeled tumor regions and scales the width and height with a factor from 0.5 to 1. Scaled tumor regions were randomly placed in the original image near the spinal region. The detection model was divided into a feature extraction network (FEN), feature pyramid network (FPN) [12], region proposal network (RPN), and region of interest (ROI) extraction module. The FEN extracted image features which may contain tumor information, using ResNeXt101 [13] as the backbone network, which is an upgraded version of ResNet101. We also added deformable convolution [14] to ResNeXt101 to adapt it to various shapes of the tumor regions. Five scales including 1/4, 1/8, 1/16, 1/32, and 1/64 of the original image were used to extract different receptive field feature information, as shown in Fig. 3, and the number of feature maps was 128, 256, 512, 1024, and 2048, respectively.

**Fig. 4:**
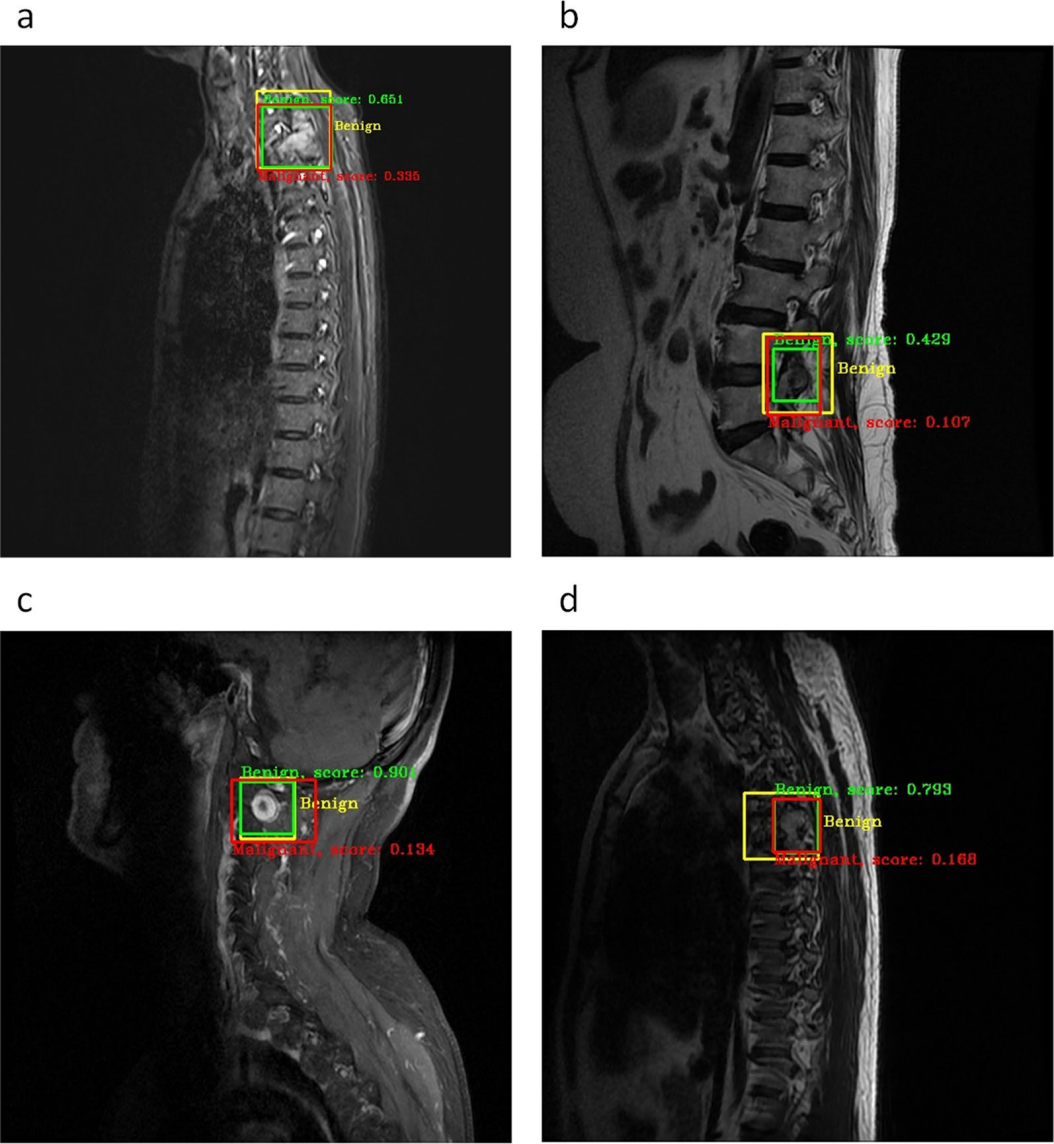
Tumor regions detected and rough classification results

The FPN was used to fuse the five different scale features. Subsequently, the RPN generated a certain number of candidate boxes that may contain tumors, and the ROI adjusted the size of the selected candidate boxes to identify the tumors as benign or malignant. Non-maximum suppression (NMS) [15] was used to determine the final location of the tumor and the probability of being benign or malignant. Figure 4 shows the results of the proposed detection model. The green boxes and labels indicate the benign tumor and its probability, respectively, the red boxes indicate the malignant tumor, and the yellow boxes indicate the ground truth.

### Sequence classification model for benign and malignant diagnosis

The tumor detection model locates and roughly identifies tumor regions of every image from the same patient, which may result in false positives. Continuous frames contain more contextual information, which is useful for accurate diagnosis. Images in each sequence correspond to a continuous tumor region; therefore, we proposed a sequence classification model based on ResNeXt101 to further classify benign or malignant tumors.

In the training stage, we selected the largest labeled tumor region in the sequence and obtained N continuous regions with this size and location as the tumor region of all images in the whole sequence, and then rescaled the size to $112\times 112\times N$ pixels. Extraction was repeated if the labeled images in the sequence were less than N. To expand the training data, there was a 50% probability of randomly extracting images with tumor regions and a 50% probability of extracting images according to the index of Digital Imaging and Communications in Medicine (DICOM). The different sample rates were used to maintain a balance between benign and malignant samples during training, which can prevent the model from overfitting a certain tumor category. In the testing stage, based on the detected tumor regions from the above tumor detection model, we selected the largest detected tumor region and obtained N continuous regions of this size and location as the tumor region of all images in the whole sequence. The size was rescaled to $112\times 112\times N$ pixels. Multiple adjacent tumor regions of the sequence were used as the input, and the probability of a benign or malignant of the sequence was the output from the sequence classification model.

### Age information for benign and malignant diagnosis

**Fig. 5:**
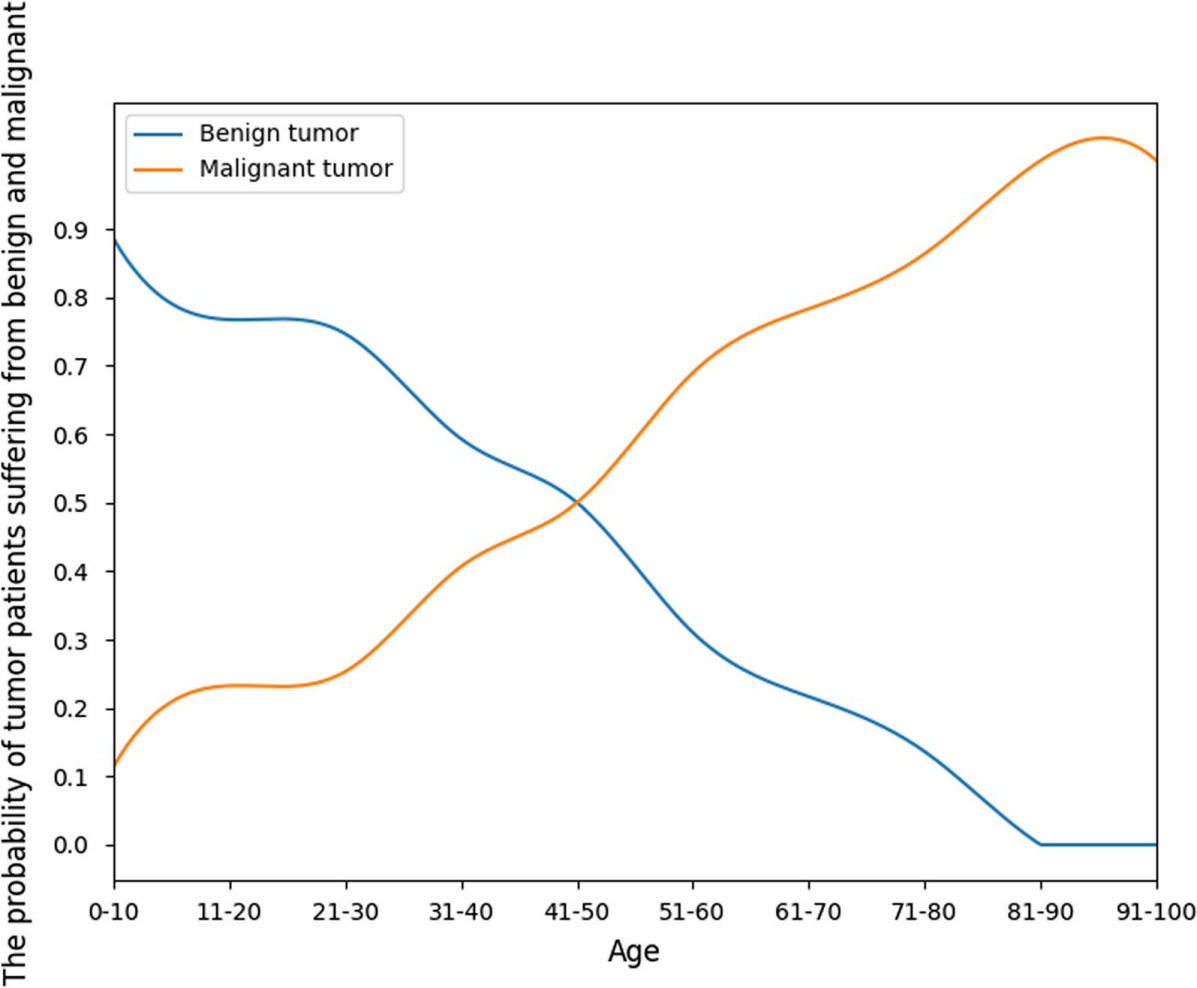
The probability of benign and malignant tumors with different ages

We determined the relationship between the probability of malignant or benign tumors and the age of each patient in our training set. Figure 5 shows that the probability of malignancy increased with age, and the probability of malignancy generally increased to approximately 50% over the age of 40 years and almost 100% over the age of 80 years. We used the statistical probability of benign and malignant tumors in different age groups as a reference for patient-level diagnoses.

### Multi-model weighted fusion strategy

To further improve the diagnostic performance for benign and malignant tumors, we proposed a multi-model weighted fusion strategy, as shown in Eq. (1).

1 $$P_{i}^{j,p} = \lambda_{1} \times D_{i}^{j,p} + \lambda_{2} \times M_{i}^{p} + \lambda_{3} \times A_{p}$$

where $P_{i}^{j,p}$ represents the final benign and malignant probabilities of the *j*-th image of the *i*-th sequence, where $D_{i}^{j,p}$ represents the probability from the tumor detection model with the *j*-th image of the *i*-th sequence of the patient, $M_{i}^{p}$ represents the probability from the sequence classification model with the *i*-th sequence of the patient, and $A_{p}$ represents the probability based on the patient’s age. $\lambda_{1} , \lambda_{2} , \lambda_{3}$ are the weights of the three terms.

The benign and malignant tumor categories of all images in each sequence were obtained by using Eq. (1), and the category with the largest proportion was selected as the sequence category. Finally, the category with the largest proportion of all sequences was selected as the benign or malignant category for this patient.

### Metrics

All the models were trained on an Intel E5-2640 CPU and an NVIDIA GTX1080Ti GPU. Samples of malignant tumors were considered positive. Area under the curve (AUC) [16], accuracy (ACC), sensitivity (SE), and specificity (SP) were used as evaluation metrics. ACC, SE, and SP are defined in Eqs. (2), (3), and (4), respectively. It should be noted that our task was to diagnose tumors based on early images of patients. This is a classification task that uses deep learning. The AUC, ACC, SE, and SP are the common metrics used to measure the classification effect. Evaluation methods such as RECIST are not applicable to our task.

2 $${\text{ACC}} = \frac{{{\text{TP}} + {\text{TN}}}}{{{\text{TP}} + {\text{FN}} + {\text{TN}} + {\text{FP}}}}$$

3 $${\text{SE}} = \frac{{{\text{TP}}}}{{{\text{TP}} + {\text{FN}}}}$$

4 $${\text{SP}} = \frac{{{\text{TN}}}}{{{\text{TN}} + {\text{FP}}}}$$

To show the diagnostic level of radiologists, spine surgeons, and our model at the same time, we invited three doctors to make a diagnosis based on the images and age information of patients in the test set, including one radiologist (D1: 18 years’ experience) and two spine surgeons (D2: 24 years’ experience, D3: 8 years’ experience).

## Results

### Comparison of different fusion strategies

We compared the results of the six different fusion strategies on the test set. In our experiments, *N* = 16 for the sequence classification model was better than *N* = 4 or *N* = 8. The results of the different fusion strategies are shown in Table 3, where Det, Seq, and Age represent the tumor detection model, sequence classification model, and age statistical information, respectively. The different strategies correspond to the different *λ* values in Eq. 1*.* For example*,* $\lambda_{1} = 0.45$, $\lambda_{2} = 0.45$, $\lambda_{3} = 0.1$ for Det-Seq-Age, $\lambda_{1} = 0.45$, $\lambda_{2} = 0$, $\lambda_{3} = 0.1$ for Det-Age, and $\lambda_{1} = 0.45$, $\lambda_{2} = 0$, $\lambda_{3} = 0$ for Det. All fusion strategies are based on the tumor region detected by the detection model.

**Table 3:** Benign and Malignant tumor prediction results with different fusion methods on the test set

Fusion method	ACC	AUC	SE	SP
Det	0.721	0.733	0.500	0.844
Det-Age	0.736	0.738	0.500	0.867
Seq	0.693	0.753	0.660	0.711
Seq-Age	0.693	0.751	0.660	0.711
Det-Seq	0.800	0.830	0.740	0.833
**Det-Seq-Age**	**0.821**	**0.839**	**0.720**	**0.878**

The bold highlight the relatively good results

As shown in Table 3, the Det-Seq fusion strategy (ACC: 0.800, AUC: 0.830) was better than the detection model-only method Det (ACC: 0.721, AUC: 0.733) and sequence classification-only model Seq (ACC: 0.693, AUC: 0.753). In addition, after considering age information, the results of Det-Seq-Age showed significant improvement (ACC: 0.821, AUC: 0.839) for benign and malignant tumor diagnosis.

### Comparison between WFF and doctors

Table 4 shows the comparison results of the WFF and three doctors. “MRI” indicates that the doctors did not refer to age information, but only referred to MRI images. “MRI-Age” indicates that the doctors referred to age information and MRI images. The “Avg. Time” represents the average time that the doctor or model spent diagnosing a patient, that is, the time between the model and doctor seeing the images and making the diagnosis result. The average diagnosis time of the WFF for each patient was less than one second, which is much faster than that for all doctors. Compared to D1, D2, and D3, the ACC of the WFF without age information improved by 5%, 13.6%, and 18.6%, respectively. The ACC of the WFF with age improved by 13.5%, 8.5%, and 18.5%, respectively. It should be noted that the ACC of D2 and D3 improved after referring the age information but decreased for D1 after referring to the age information because of paying too much attention to age. The SE and SP of WFF were both higher than those of D1 and D2. Although D3 had a higher sensitivity (92.0%) without age information, his ACC (61.4%) and specificity (44.4%) were lower.

**Table 4:** Comparison between WFF and three doctors for benign and malignant tumor prediction

Method	Avg. time(s)	ACC	SE	SP
D1 (MRI)	35.44	0.750	0.660	0.800
D2 (MRI)	51.68	0.664	0.580	0.711
D3 (MRI)	47.62	0.614	0.920	0.444
**Det-Seq**	**0.850**	**0.800**	**0.740**	**0.833**
D1 (MRI-Age)	41.75	0.686	0.700	0.678
D2 (MRI-Age)	27.26	0.736	0.720	0.744
D3 (MRI-Age)	37.41	0.636	0.880	0.500
**Det-Seq-Age**	**0.742**	**0.821**	**0.720**	**0.878**

The bold highlight the relatively good results

### Comparison of different vertebral locations

**Fig. 6:**
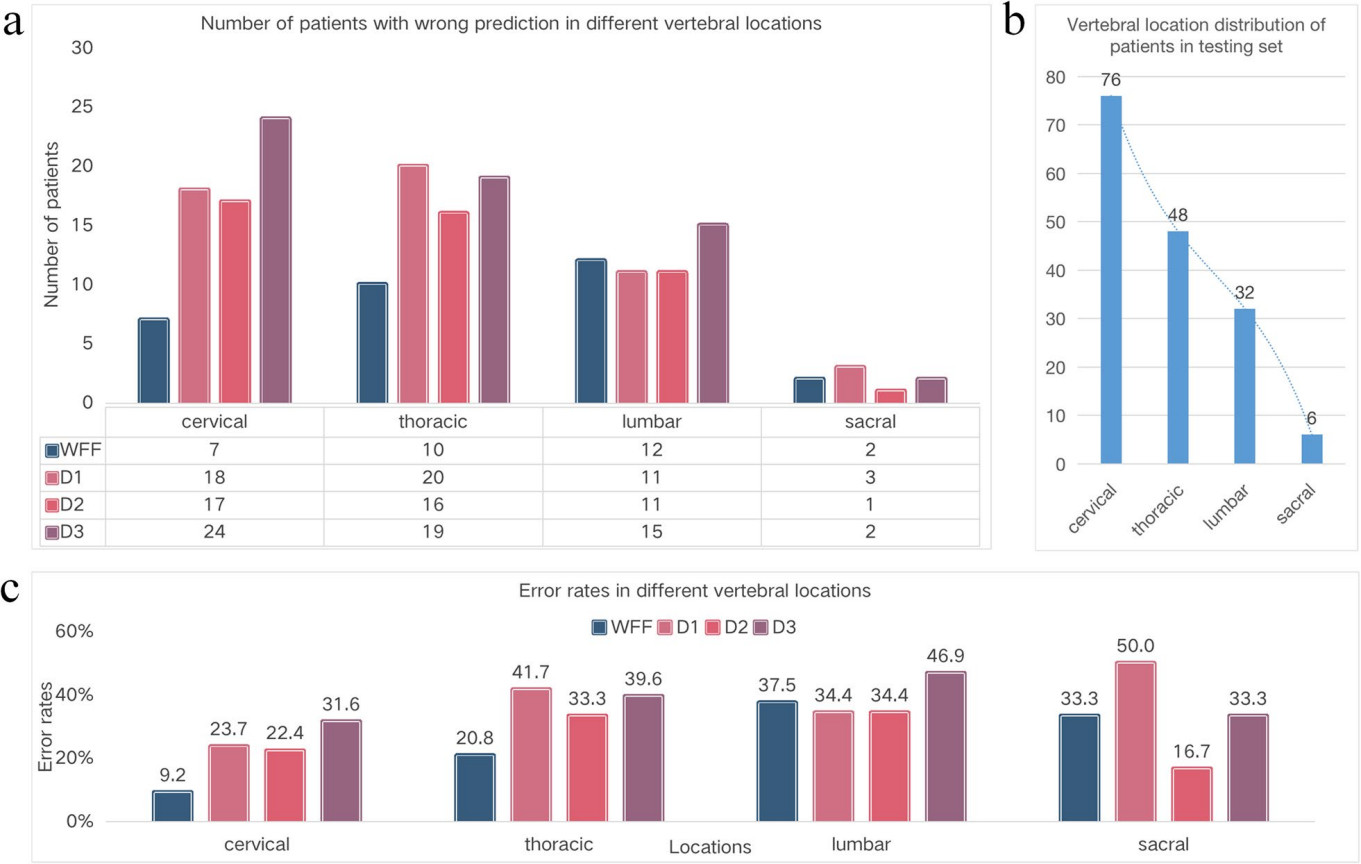
**a** Number of patients with wrong prediction in different vertebral locations. **b** Vertebral location distribution of patients in the testing set. **c** Error rates in different locations

To further explore the difference between WFF and doctors, we counted the number of patients with incorrect predictions, the error rate in different vertebral locations, and the distribution of vertebral locations in the testing set, as shown in Fig. 6.

The number of patients with incorrect prediction and error rate by WFF in most locations was lower than that of the doctors. As shown in Fig. 6a, D2 and D3 had the largest incorrect predictions at the cervical vertebra, D1 had the largest number at the thoracic vertebra, and WFF had the largest number at the lumbar vertebra, while both WFF and doctors had the lowest number at the sacral vertebra. By observing the number distribution in different vertebral locations in Fig. 6b, it can be seen that the number of patients with tumors in the cervical and thoracic vertebrae was large, and the misprediction trend of doctors was consistent with the location distribution, however, the trend of WFF was opposite. The reason for this phenomenon is that for the deep learning model, the more samples, the better the diagnosis effect, which shows that for the same vertebral location, the model can surpass doctors through the learning of a large number of samples.

However, as shown in Fig. 6c, the error rate trend of WFF and doctors is different from that in Fig. 6b; most doctors and WFF have a lower error rate in the cervical vertebrae and the highest error rate in the lumbar vertebrae. Our test set represented the distribution of the overall data of the cooperative hospital. The reason for this phenomenon is that both WFF and doctors need to accumulate experience from a large number of cases. The more cases, the richer the experience and the lower the error rate. This shows that for the deep learning model, more representative samples can help improve its diagnostic performance.

### Comparison of different sequences

**Fig. 7:**
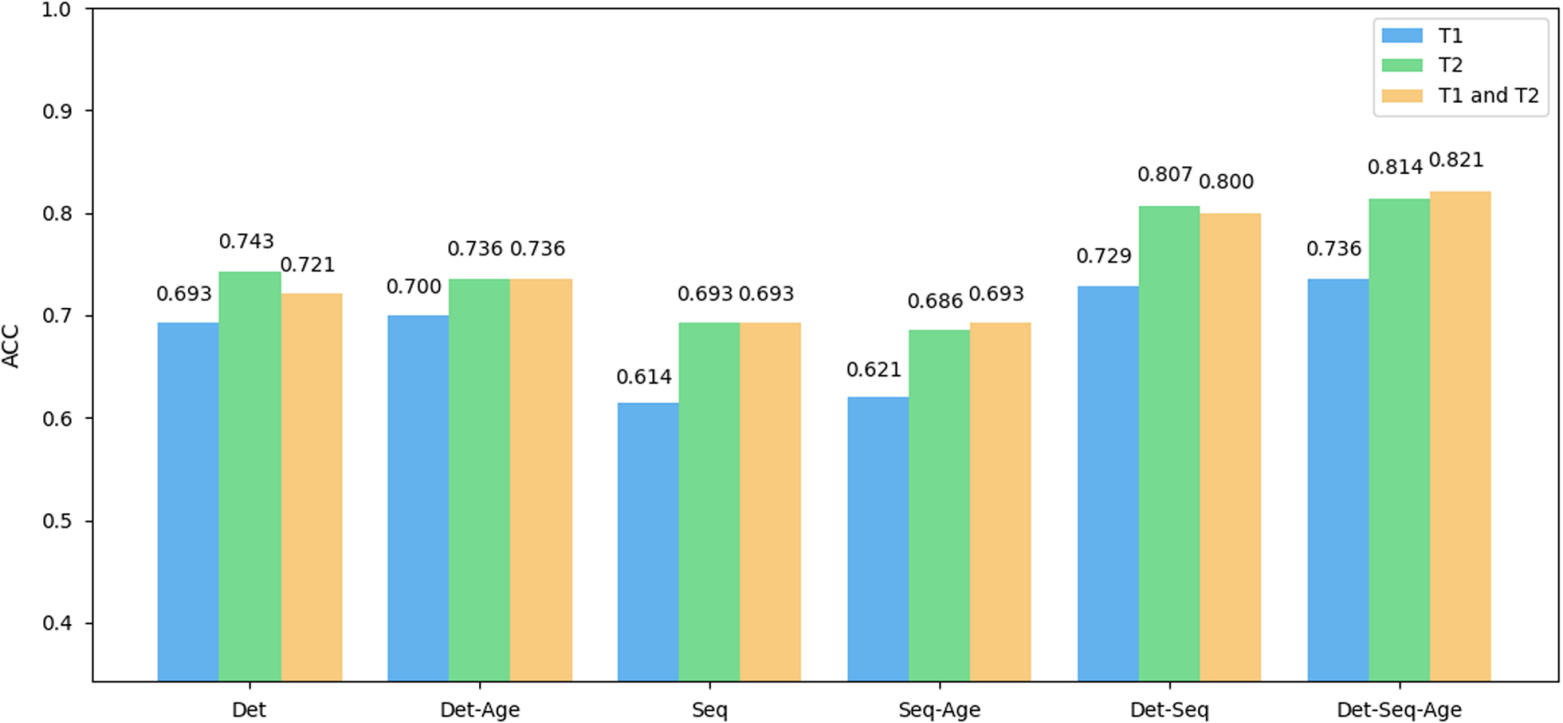
The ACC of different fusion methods based on T1, T2, and T1&T2 sequence

The above results were obtained by using all sequences of patients, including T1 (T1WI) and T2 (T2WI and FS-T2WI). To verify which sequence had the greatest impact on the final result, we further obtained ACC with only T1 (T1WI) or T2 (T2WI, FS-T2WI) sequences on the test set. For the six fusion methods, as shown in Fig. 7, the ACC of the T2 (T2WI, FS-T2WI) sequence showed an improvement of approximately 3% to 8% compared to that of the T1 (T1WI) sequences, and the results of combining T1 (T1WI) and T2 (T2WI, FS-T2WI) sequences were similar to those obtained using only T2 (T2WI, FS-T2WI) sequences. This shows that the T2 (T2WI and FS-T2WI) sequence is more helpful for tumor diagnosis in artificial intelligence models. However, the proposed WFF is not limited to specific scanning images, such as T1 (T1WI) and T2 (T2WI, FS-T2WI). When there are enough samples, it is also applicable to other images, such as post-contrast images, or even the combination of a variety of different images.

## Discussion

There have been several studies conducted about spinal tumors, which are similar to the present study. For example, Hammon et al. [17] developed an SVM model to detect spinal metastases based on CT images of 114 patients, and similar work was undertaken by O’Connor et al. [18]. In addition to directly identifying tumor categories, Burns et al. [19] used a segmentation method to detect tumor regions based on images of 49 patients, and then used SVM to identify these tumor regions. Chianca et al. [20] used hCAD and PyRadiomics tools to extract image features and then used the machine learning method to select and recognize features based on single-frame images to identify benign and malignant spine tumors of 146 patients with the lesion region annotated by the doctor. Wiese et al. [21] proposed an automated method based on a watershed and graph-cut algorithm to detect spinal metastases in CT images. Yao et al. [22] further proposed an SVM-based algorithm to improve initial detection using the watershed algorithm for the detection of spinal metastases in CT images. The aforementioned methods use traditional feature extraction and machine learning methods on small case scales.

In recent years, with the development of deep-learning technology, an increasing number of new technologies have been used in the field of medical imaging, including lesion segmentation, detection, and classification. U-Net series models are usually used to segment lesions from CT or MRI [23–26]. The detection models represented by Faster-RCNN have been used to detect the location of lesions in medical images [27–30]. In addition, some studies have applied deep-learning technology to lesion classification [31–34]. For example, Lang et al. [35] used the normalized cut algorithm to generate a 3D tumor mask, extracted histogram and texture features from multiple adjacent image frames used as the input of CNN and convolutional long short-term memory for differentiating metastatic lesions in the spine originating from primary lung and other cancers, which based on a dataset containing 61 patients with tumor regions annotated by doctors. Roth et al. [36] used a deep convolutional neural network as the 2^nd^ stage to refine the lesions from the 1^st^ stage from CT images for the detection of spinal metastases. Zhang et al. [37] proposed a two-step pipeline containing a Faster-RCNN to detect abnormal hyperintensity and an RUSBoost classifier to reduce the number of false-positives based on 121 patients, of which 73 were for training, and 48 for validation. Liu et al. [38] used six classifiers to distinguish between HRC and non-HRC statuses based on 89 patients with multiple myeloma using MRI. The above methods were evaluated in small-scale cases, most of which were directly analyzed on manually marked tumor regions without automatic tumor detection processing, and some did not use clinical information.

In contrast to the above methods, this paper proposes a multi-model weighted fusion framework (WFF) based on deep learning to diagnose benign or malignant spinal tumors by using patient sagittal MRI sequences and age information, which can automatically detect the tumor location and make patient-level benign and malignant diagnosis based on all sequences of patients. Doctors usually refer to clinical information to diagnose spine tumors, such as age information. For example, the older the patient, the greater is the probability of a malignant tumor. However, this conclusion is not absolute, as there are still some younger patients with malignant tumors. Therefore, the accuracy of doctors may improve or decrease after referring to age information, while WFF can well adjust the relationship between age information and model results, which may avoid misleading the fusion results. The experimental results demonstrate the effectiveness of the proposed WFF.

### Limitations

The retrospective study design would have resulted in inevitable bias, and all data were collected from a single center, thereby limiting the sample size of the study. In the proposed method, the detection and classification of tumor regions in each image from the sequence is the basic and key technology for patient-level diagnosis. To improve the recall rate of tumor regions, the tumor detection model produces a certain number of false-positive regions, which would greatly complicate patient-level fusion. At the same time, the visual features of some benign and malignant spine tumors are not obvious, making it difficult to distinguish between benign and malignant tumors in some situations. Therefore, improving the performance of the detection model, reducing the false-positive rate, and improving the classification accuracy of the model will be the focus of future research. In addition, owing to the limitations of the experimental conditions, this study only used age information. If more clinical information is available, it is believed that diagnostic performance will be improved.

## Conclusions

Owing to the rich tissue sources, pathological types, and diverse clinical symptoms, diagnosis of early benign and malignant spine tumors from MR images is very difficult, even for medical experts. This study proposes a multi-model weighted fusion framework (WFF) based on deep learning that combines both medical images and age information. The experimental results demonstrate the effectiveness of the proposed WFF.

## Data Availability

All information is included in this manuscript.

## Abbreviations

ACC: Accuracy
AUC: Area under the ROC curve
DICOM: Digital Imaging and Communications in Medicine
FEN: Feature Extraction Network
FPN: Feature Pyramid Network
IRB: Institutional Review Board
MRI: Magnetic Resonance Imaging
NMS: Non-maximum Suppression
ROI: Region of interest
RPN: Region Proposal Network
SE: Sensitivity
SP: Specificity
WFF: Weighted Fusion Framework
